# Ethical oversight in quality improvement and quality improvement research: new approaches to promote a learning health care system

**DOI:** 10.1186/s12910-015-0056-2

**Published:** 2015-09-17

**Authors:** Kevin Fiscella, Jonathan N. Tobin, Jennifer K. Carroll, Hua He, Gbenga Ogedegbe

**Affiliations:** Department of Family Medicine, University of Rochester Medical Center, Rochester, USA; Clinical Directors Network (CDN), New York, NY USA; Albert Einstein College of Medicine of Yeshiva University, Bronx, NY USA; The Rockefeller University Center for Clinical and Translational Science, New York, NY USA; Department of Family Medicine, University of Colorado, Denver, USA; Department of Biostatistics, University of Rochester Medical Center, Rochester, NY USA; Department of Population Health, Center for Healthful Behavior Change, New York University Langone Medical Center, New York, NY USA; Family Medicine Research, 1381 South Ave, Rochester, NY 14620 USA

**Keywords:** Ethics, Research, Research subjects, Ethics, committees research, Health services research, Quality improvement, Quality improvement research, Ethics, clinical, Informed consent, Waiver of informed consent, Learning health care systems

## Abstract

**Background:**

Institutional review boards (IRBs) distinguish health care quality improvement (QI) and health care quality improvement research (QIR) based primarily on the rigor of the methods used and the purported generalizability of the knowledge gained. Neither of these criteria holds up upon scrutiny. Rather, this apparently false dichotomy may foster under-protection of participants in QI projects and over-protection of participants within QIR.

**Discussion:**

Minimal risk projects should entail minimal oversight including waivers for informed consent for both QI and QIR projects. Minimizing the burdens of conducting QIR, while ensuring minimal safeguards for QI projects, is needed to restore this imbalance in oversight. Potentially, such ethical oversight could be provided by the integration of Institutional Review Boards and Clinical Ethical Committees, using a more integrated and streamlined approach such as a two-step process involving a screening review, followed by a review by committee trained in QIR. Standards for such ethical review and training in these standards, coupled with rapid review cycles, could facilitate an appropriate level of oversight within the context of creating and sustaining learning health care systems.

**Summary:**

We argue that QI and QIR are not reliably distinguishable. We advocate for approaches that improve protections for QI participants while minimizing over-protection for participants in QIR through reasonable ethical oversight that aligns risk to participants in both QI and QIR with the needs of a learning health care system.

## Background

In a provocative pair of articles, Kass et al. and Faden et al. argue that the research-treatment distinction is no longer tenable, and propose new ethical principles for guiding both research and treatment in the context of a learning health care system [1, 2]. Their proposed principles regard the failure to use information gathered from the point of care for research and learning as unethical. However, their papers leave open the question of how to begin such reform. Actionable next steps under current regulations are uncertain.

Our experience with many research and quality improvement research projects has prompted us to reflect on these complex issues and propose concrete next steps that might be adopted under the current ethical regulations for research. We focus on the interface between quality improvement research (QIR) and quality improvement (QI). We argue that QIR and QI are indistinguishable; that they are sufficiently distinct from other types of clinical research that they warrant their own distinct processes and committees for ethical oversight and that the level of oversight should balance risk to participants with an ethical imperative to improve care.

## Discussion

### Existing guidelines distinguish QI and research

The United States Department of Health and Human Services (DHHS) regulates biomedical and behavioral research ethics in the United States under the Common Rule. It has remained largely unchanged since 1991 and reform appears stalled [3]. Both the Common Rule and World Health Organization (WHO) guidelines attempt to distinguish QI and research [4]. This is understandable based on concerns that placing QI under The Common Rule could hinder processes designed to improve patient care and ultimately reduce patient risk [5].

### QI and QIR are not reliably distinguishable

The Common Rule, WHO guidelines, and consensus statements have sought to distinguish research and QI based on the rigor of methods (internal validity) and generalizability (external validity) of the findings [4–6]. On close inspection, these criteria alone do not hold up.

### Rigor of methods

Traditional research methods, and the methodological rigor they represent, enhance the internal validity of the findings whether considered research or not. That is, they improve confidence that the findings are valid for the population(s) under scrutiny. However, high internal validity is not unique to research including quality improvement research (QIR). Rather, use of rigorous methods is based on two often competing goals. The first goal is minimizing type I error (essentially a false positive finding or inferring an effect exists when none actually exists) as well as type II (a false negative finding or inferring no effect exists when one actually exists). The second goal is feasibility of the methods. Feasibility issues include technical, methodological (e.g. types of design) operational (e.g. effects on workflow), costs, and acceptability to patients, providers, administrators, and communities. The desire to reduce invalid inference, whether due to type I or II errors, applies to both QI and QIR. For example, a large, national health plan conducting QI might have a strong desire to minimize type I error through use of highly rigorous methods, including randomization, while also minimizing type II error, through sufficiently large sample sizes, before rolling it out to all plan members. As with QIR, QI must balance strong methods against feasibility. The competing goals of getting the correct answer with practical constraints do not clearly distinguish QI and QIR.

The risks associated with invalid findings, whether QI or QIR, have important ethical implications. Both entail costs and potential burden to participants, and the possibility of risks to future patients. Use of less rigorous study designs, i.e. those associated with a higher risk of confounding, may result in spurious findings and invalid inferences. Furthermore, this means that research and/or health care resources are potentially squandered and that participants are exposed to unnecessary risks and burdens (however minimal) with the possibility of little or no benefit, or even the possibility of harm, particularly where understudied subgroups experience paradoxical effects.

### Generalizability of findings

The second major criterion −generalizability (external validity)- is also problematic in distinguishing QI from QIR. First, QI and QIR (and other pragmatic research) often involve use of “real world” settings and participants. In contrast, traditional clinical research is often much less generalizable, as it is often conducted in highly specialized academic settings, and with highly selected (homogeneous) patient populations that exclude many comorbid conditions [7, 8]. A traditional randomized controlled trial with a long list of highly selective eligibility criteria may yield findings that are less generalizable than a QI intervention conducted with patients with fewer or no eligibility restrictions, particularly those that exclude patients with multiple comorbidity. Traditional randomized controlled trials are often intended to generate generalizable knowledge of a different sort, i.e. scientific principles, mechanisms, processes or theory. Nonetheless, in terms of extrapolation of findings regarding the intervention itself, these trials often have poor generalizability [9]. In contrast, QIR trials often examine patient populations similar to those used in QI projects. In both cases, the selection of participants is based on the level of generalizability, i.e. to other patients in a system or more broadly to similar participants.

The second challenge to generalizability as a distinguishing feature is that generalizability represents a broad continuum without clear lines of demarcation. Most QI findings are intended to generalize beyond the participants, health care delivery systems and practice settings involved in the QI project. A large health plan or large hospital system might conduct a quality improvement project within several geographic regions with the hopes of generalizing the findings nationally. Depending on the context and unique features of the organization, the findings may or may not be generalizable to other plans and practice settings. Similarly, few QIR projects involve sufficiently large or diverse population of patients, of clinicians and of practices to ensure widespread generalizability and replicability.

The generalizability conundrum is not remedied by resorting to the stated intent of the project. Asking the project leaders whether they intend to generalize their findings (i.e. knowledge gleaned) beyond the participants is misleading. QI projects invariably involve some degree of generalizability, even if it is limited to the population served by the institution that generates the findings from a sample of observed patients. This may include extrapolation of findings to concurrent non-participating patients or to future patients. In contrast to QI leaders, QIR leaders often overestimate the generalizability of their findings and minimize threats to external validity [10]. In addition, stated intent may be distorted to reflect the desire of expediency based on avoiding IRB review or based on journal requirements for IRB review. In sum, generalizability is a continuum without clear cut-offs that reliably distinguish QI for QIR.

### Other criteria

Other proposed criteria for distinguishing QI from research similarly do not reliably distinguish QI and QIR. Casarett et al. suggest that a project should be deemed research if a majority of patients involved are not expected to benefit [11]. These criteria are more helpful in distinguishing QI from traditional clinical research than from QIR, which potentially entails as much direct benefit to participating patients as QI projects. Given the absence of standards for implementing QI, there is no *a priori* reason to believe that expected benefit would be greater with QI than with QIR.

Whether a project imposes additional risks and burden to improve generalizability represents a second criterion proposed by Casarett et al. [11] It, too, fails the test. Pragmatic research, including QIR, strives to avoid undue burden on participants [8]. Other proposed criteria, including source of funding, plans for publication and dissemination, and involvement of researchers in the project [12], are arbitrary and not grounded in ethical principles relevant to protection of participants, and may be subject to change over time as a project evolves and as new questions are raised based on information gleaned from the project.

### Balancing competing ethical principles

If QI and QIR are not readily distinguishable, then what are the implications for ethical oversight? How should ethical oversight be balanced with the need to create continuous learning health care systems? When is informed consent by patients necessary and under what conditions are informed consent required from clinicians and other staff? With the increased attention to “pragmatic research” using electronic health records, under what conditions is a waiver of informed consent necessary and reasonable? In the remainder of this paper, we address these questions.

### Risk to participants versus the imperative to improve care

The level of scrutiny should balance the risk to participants with the need to expedite care improvement including an ethical imperative to learn quickly [13, 14]. This balancing of competing ethical principles should be the primary task, rather than seeking to resolve an arbitrary distinction between QI and QIR. Currently, most large QI projects appear to undergo some level of review, particularly with regards to participant risk and confidentiality of data [15]. However, the quality of this review may be variable and typically ill defined [15]. Length of IRB reviews among the same QI and QIR projects vary widely [16–18]. Standardization of oversight for QI and QIR is needed. This approach would help prevent future mishaps, e.g. suspensions of national QIR projects [19].

### Ethical review considerations

Unlike current QI reviews (which are often internal) [15], ethical oversight reviews including those for QI should be independent and should reflect bioethical and methodological expertise, and perspectives from administrators, clinicians and patients. Ethical review should consider soundness of the methods, including whether the methods are sufficiently rigorous to meet the goals and scope of the project and minimize invalid inferences. An ethical review for QI and QIR would not preclude use of rapid cycle or Plan-Do-Study-Act (PDSA) approaches that are fundamental to learning health care systems, but would help to ensure appropriate use of resources, as well as protection of patient privacy and safety.

Ethical oversight can be provided using current institutional oversight committees with some modifications. This would mean creation of a separate committee to review QI/QIR projects. Provided that current ethical guidelines for research, e.g. ‘The Common Rule” are followed, and that reasonable criteria for minimal risk criteria are applied, QI and QIR could be reviewed by the same committee. Burden could be further reduced using a two-step process.

### Two-step process

A two-step process minimizes delays and the burdens for review for minimal risk projects while ensuring documentation of minimal review. The first step would involve submission of a succinct, e.g. one page document that summarizes key ethical considerations. An Alberta, Canada Consensus Initiative has developed ethical criteria for consideration for QI projects [20]. A children’s hospital in Australia has taken a similar approach to QI [21]. These criteria, which are also relevant to QIR, include usefulness of knowledge, methods, fair appropriate selection of participants (or data), steps to maximize benefit and minimize risk, respect for rights of individuals, communities and populations, and informed consent. These criteria are similar to those used to determine IRB exemption.

The QI/QIR document would be reviewed by a person designated by the oversight committee (first step). This reviewer would either approve the project or refer the project for further committee review (second step). Optimally, this would occur within a few days and not take longer than a week for a determination. This two-step process is similar to “expedited reviews.” Potential differences would include a shorter application, shorter turn-around times, and review by members with QI/QIR ethics expertise.

Periodic auditing of documents and decisions by a second member of the oversight committee could help to standardize approval, referral decisions and documentation, while ensuring quality oversight through an audit trail. The first quick step minimizes the risk of projects being considered “exempt” from full review in the absence of auditable documentation of minimal, independent review.

### Integration of clinical and research ethics oversight

Who should provide ethical oversight of QI/QIR? QI/QIR are sufficiently distinct from other types of clinical research that they warrant their own dedicated oversight committee. In theory, an oversight committee could reside within an existing IRB structure, i.e. as a QI/QIR IRB committee. Alternatively and regulations permitting, it could represent a new organizational structure. Expertise on this oversight committee would optimally include patients, including previous participants in QI/QIR, and experts in conducting QI and QIR, in addition to experts in ethical oversight. Improved integration of ethical oversight within the context of a learning health care system could be achieved by expanding committee membership beyond members with expertise in applying ethical principles to groups, to include members with expertise in applying ethical principles to individual patients. This could be accomplished by inviting members from Clinical Ethical Committees (CECs) to join the QI/QIR ethical oversight committee. CECs provide clinical consultations not only to clinicians, staff, patients, and families, but also establish and oversee institutional guidelines and procedures that involve ethical concerns [22]. CECs are experienced in weighing risks and benefits during clinical care and thus could potentially offer a valuable perspective in applying ethical principles relevant to a learning health care system in the context of QI/QIR.

This integration of CECs into ethical oversight of QI/QIR would minimize the silos between research and treatment and facilitate cross-fertilization of expertise and perspectives. An integrated committee might provide more informed oversight of QI/QIR than IRBs with traditional membership.

### Training of QI/QIR ethical oversight committees

Even among traditional IRBs, there is wide variation in IRB’s composition, efficiency, perspectives, training and requirements [23]. Members in a QI/QIR oversight committee will require relevant training. Ideally, training would be provided conjointly with other members in order establish shared understanding of review criteria. Most patients have not been trained in ethical oversight, and are less familiar with QI/QIR ethical issues than non-patients. However, even many experts in QI have not been formally trained in ethical principles. Members of CECs will need to learn to apply ethical principles in the context of QI and QIR. Traditional IRB members will need to learn how to weigh protections to patients in the context of an imperative to continuously improve health care.

Training would be similar to that adopted by A Project Ethics Community Consensus Initiative (ARECCI) for review of quality improvement projects [20]. The training would include selected readings followed by facilitated group discussion. Key topics would include: 1) Purposes and appropriate methods of QI/QIR; 2) Specific examples of ethical concerns; 4) Issues related to consent and conditions required for a waiver of informed consent; 5) Process for balancing competing ethical concerns, including potential benefits, risks, and burdens to potential participants, i.e. patients, staff and/or administration) and to the health care system and patients at large. Most notably, such training would aim to establish shared understanding among committee members regarding how to balance competing ethical concerns in ways that would facilitate rather than hinder the development of learning health care systems. Shared understanding and common goals are critical to ensuring an efficient, timely and fair review. For example, the committee would decide under what conditions the ethical benefit of ensuring that project findings are more generalizable (by granting a waiver of informed consent) trumps the need for obtaining individual consent from all participants. Similarly, the committee would decide when more rigorous methods are required to avoid erroneous inferences followed by roll-out of a flawed quality improvement intervention.

This new committee would also establish a roadmap for integrating committee self-assessment, user feedback, and review into the operations. This change in paradigm will not occur overnight. It will require an iterative process involving action, reflection, and revision by the committee. It will require careful attention to process over time including timeliness of reviews, useful revisions to projects, and input from multiple perspectives and stakeholders including patients. Most importantly, it will require openness to new thinking, new ways for ethical review, and engagement of clinicians and patients in oversight.

## Informed consent

Reluctance to subject QI to ethical oversight reflects a legitimate concern that such ethical oversight could undermine creation of learning health care systems. [6] This includes concerns about problematic delays in implementing QI, but also concerns about the potential for requiring informed consent for participants in QI projects, and the likelihood of introducing selection bias by those who opt-in versus opt-out [24]. These are valid concerns; there is an ethical obligation on the part of patients, staff, and clinicians to contribute towards a learning health care system, with benefits that accrue not only to the patients, but also to society as a whole [2].

Informed consent is intended to protect participants from exposure to additional risks posed by ‘research’ they have not agreed to that goes beyond the standard of clinical care, while respecting their autonomy and preferences [25]. This process is analogous to informed consent for patient care. In both cases, informed consent balances benefits, risks and practical considerations. For minimal-risk procedures, such as commonly ordered blood tests during patient care, informed consent from patients is neither required nor routinely obtained beyond general consent for treatment. Obtaining informed consent for every single test could result in greater harm (through delays and wasted resources) than benefit. Formal informed consent is reserved for procedures where the stakes, both risks and benefits, are greater e.g. surgical procedures or medications that carry significant side effects [26]. However, when the procedure entails appreciate risk such as surgical treatment, informed consent specific to the procedure is obtained from the patient. This same balance between benefits, risks and practical considerations is relevant to both QI and QIR.

Under current USA Federal Regulations informed participant consent may be waived if the research in question poses “no more than minimal risk to the participants,” and if failing to obtain informed consent “will not adversely affect the rights and welfare of the subject,” and if “the research could not practicably be carried out,” and if “whenever appropriate, the subjects will be provided with additional pertinent information after participation.” [27] These considerations are applicable to virtually all QI and much of QIR though the ultimate determination lies in the oversight committee's assessment of competing risks and benefits. For example, it may not be feasible to conduct a pragmatic trial of outreach for patients past due for cancer screening using traditional informed consent. The primary target population for these efforts would be systematically excluded by a requirement for informed consent, because this population is the most difficult to reach due to changes in address, phone numbers and infrequent office visits [28, 29]. Thus, results would not be generalizable to the population of interest. In this case, we agree with Faden et al. that a waiver of informed consent is necessary [30].

Individual informed consent is often not feasible for cluster randomized controlled trials and comparative effectiveness research (CER) studies where the organization is the primary target of the intervention [31, 32]. Requiring individual informed consent from all participants including all patients render these trials obsolete with accompanying loss of new knowledge. Obtaining waivers for individual informed consent in cluster randomized trials, requires consideration of feasibility, knowledge gained, and the balance of benefits and risks to participants [31, 33, 34].

Informing new patients upon their enrollment in care regarding potential QI/QIR and obtaining acknowledgement, i.e. universal consent, represents another way to inform patients when opt-out might not be feasible. This approach could strengthen requests for waivers of informed in the presence of favorable benefit-risk to participants while offering patients with the choice to obtain care at other organizations. For higher risk projects, opt-out approaches could be used to recruit (and subsequently consent) patients. This could include notifying the patient by mail or electronically that a project has begun and that they have the right to opt-out by calling a number [35]. This passive approach to informed consent may minimize response bias that occurs with active, opt-in consent [36].

## Conclusion

QI and QIR are not reliably distinguishable, but rather comprise a continuum of activities. We recommend the development and institutionalization of new approaches that build on existing structures, such as a two-step process, to facilitate ethical review of QI/QIR and promote learning health care systems within a rapidly changing health care environment. We recommend creating expert ethical oversight committees to review both QI and QIR. Such committees can operate under many existing regulations regarding minimal risk research and waivers of informed consent. The ultimate goal of this approach is to balance the protection of the rights of participants in QI/QIR with the ethical imperative to improve care as rapidly as possible by building a healthcare system that continuously learns from its data.
